# Comparison of ultrasound contrast agent production by sonication and mechanical agitation

**DOI:** 10.1016/j.ultsonch.2026.107875

**Published:** 2026-05-06

**Authors:** Jovana Katrinka-Mavrak, Luca Bau, Jocelyne Rivera, Adam C. Sedgwick, Eleanor Stride

**Affiliations:** a Institute of Biomedical Engineering, University of Oxford, Oxford, UK; b Department of Chemistry, Kings College London, London, UK

**Keywords:** Microbubbles, Contrast agents, Theranostics, Ultrasound, Microbubble generation, Mechanical agitation, Sonication

## Abstract

Microbubbles (MBs) are surfactant-coated gas vesicles used widely in ultrasound imaging and increasingly for therapy. MBs can be produced by a range of methods, most commonly involving sonication and/or agitation. This study aimed to compare MB production by agitation with a bead mill tissue homogeniser or with a dental amalgamator, and sonication.

All methods produced MBs with a similar size distribution (10–90% inter-percentile range ∼0.44–1.6 μm), but both the MBs produced by the agitation methods had higher concentration than those produced by sonication (∼10^10^ vs ∼10^9^ particles/mL), and a more persistent ultrasound response. The lipid order of the phospholipid coating was investigated spectroscopically, and it was found that MBs produced by all three methods had a similar generalised polarisation. The effect of varying agitation frequency and duration on MB size distribution and concentration was investigated, with increases in either parameter raising MB concentration and reducing mean size, each showing distinct limiting behaviour. Lastly, the feasibility of generating larger MB volumes with the tissue homogeniser was investigated, yielding MBs of comparable size and concentration in both 2 mL and 5 mL vials. The results of this study suggest that bead mill homogenisers can generate MBs with comparable size distribution, stability and shell lipid order to those generated using dental amalgamators. At low lipid concentration (0.9 mg/mL), both methods outperform sonication; with the added advantage that there is a lower risk of contamination. The tissue homogeniser offers further advantages compared with dental amalgamators, in terms of MB production volume and reliability.

## Graphical abstract

**Figure:**
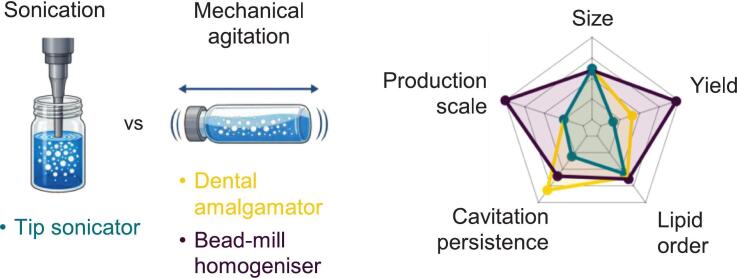

## 1 Introduction

Ultrasound imaging is a versatile and inexpensive diagnostic modality that has been routinely used in medicine for several decades. More than 50 years ago researchers discovered that the presence of gas bubbles in saline injections could transiently enhance ultrasound image contrast [1]. Since then, development of coated gas bubbles to be used as contrast agents has progressed and several formulations are now routinely used in the clinic for echocardiography and other diagnostic procedures [2]. One of the most widely used classes of contrast agents consists of phospholipid-coated microbubbles (MBs), and these are also being investigated for various therapeutic applications [3]. The phospholipid coating enhances MB stability by preventing gas diffusion and reducing interfacial tension. It also can be functionalized to enable attachment of targeting and/or therapeutic species to the bubble surface [4], [5], [6].

MBs can be produced by a range of methods. These include using low frequency (20–50 kHz) ultrasound to disperse gas in a solution of surfactant [7]. This method produces polydisperse MBs in high volumes and typically requires high surfactant concentrations (>4 mg/mL) to obtain appropriate MB yields. To address the challenge of polydispersity, various microfluidic devices have been developed for production of monodisperse MBs [8], [9], [10]. The shortcoming of this method is the substantially lower throughput compared with that obtained with mechanical agitation methods. Although both techniques are frequently used in research laboratories, their translation to the clinic has proven to be difficult because they require specialized equipment and trained operating personnel, or complex post-sample processing to preserve MB stability. Consequently, mechanical agitation has emerged as a method to produce MBs quickly and reliably and is used in both clinical and research settings [11], [12]. Repurposed dental amalgamators, which exploit large-amplitude reciprocating motion at thousands of strokes per minute, are typically used. However, these devices are limited to small batch volumes, only operate at a fixed frequency, and are prone to mechanical failure. Bead mill homogenisers, widely used for tissue homogenisation, offer similar motion profiles, with comparable frequency and amplitude. Bead mill homogenisers are structurally durable, can accommodate a broader range of vial geometries, agitate multiple vials per run, and operate at different frequencies. The aim of this study was to compare existing MB production methods with an alternative mechanical agitation method using a tissue homogeniser, with the ultimate goal of developing a more robust and scalable manufacturing protocol.

## 2 Experimental (materials and methods)

Dipalmitoylphosphatidylcholine (DPPC), 1,2-Dipalmitoyl-*sn*-glycero-3-phosphate (DPPA) and 1,2-dipalmitoyl-*sn*-glycero-3-phosphoethanolamine-N-[methoxy(polyethylene glycol)-5000] (DPPE-PEG5k) were purchased in powder form from Avanti Polar Lipids Inc. (Alabaster, Alabama, United States). Chloroform, glycerol, propylene glycol, aluminium seals for glass vials and clear glass screw top vials were purchased from Sigma-Aldrich Ltd. (Gillingham, Dorset, UK). Freeze-drying glass vials (product codes: VIA1120 and VIA1122) were purchased from Scientific Laboratory Supplies Ltd (Nottingham, UK). Glass vial rubber stoppers Fisher Scientific Ltd (Loughborough, UK). C-laurdan was purchased from Bio-Techne R&D Systems (Abingdon, UK). Perfluorobutane was purchased from F2 Chemicals Ltd (Lancashire, UK).

### 2.1 Phospholipid suspension preparation

A Definity-like formulation (0.9 mg/mL of DPPC/DPPA/DPPE-PEG5k 82:10:8 mol/mol in PBS/propylene glycol/glycerol 80:10:10 v/v) was used for all methods. 16 mg of DPPC, 1.8 mg of DPPA and 12 mg of DPPE-PEG5k were dissolved in 2 mL of propylene glycol at 55 °C for 20 min, and added dropwise to 18 mL of a 90:10 water/ glycerol mixture under vigorous stirring at 55 °C. The resulting clear suspension was stored at −20 °C and used within 1 month of preparation.

### 2.2 Microbubble production

MBs were produced either by using one of two agitation protocols, or by sonication.

- Tissue homogeniser agitation protocol:

1 mL of prepared phospholipid suspension was transferred to a 2 mL freeze-drying glass vial (internal diameter × height: 12.5 × 36.5 mm), capped with a rubber stopper and crimped with an aluminium cap. The vial was cooled on ice and connected through a 21G needle and a 3-way tap to a vacuum pump and a syringe filled with perfluorobutane (PFB). Air was replaced with PFB by 4 vacuum/refill cycles. Then, vial was inserted into a custom made insert (Fig. S4) which was placed in a Precellys Evolution tissue homogeniser (Bertin technologies, Maryland, USA). The vial was agitated at 10,000 cycles per minute (cpm) for 45 s, unless otherwise stated. Following agitation, the vial was immediately stored on ice until further use.

For experiments with larger volumes, 4 mL of the phospholipid suspension was transferred to 5 mL freeze-drying glass vials (internal diameter × height: 20 × 37.3 mm) before the gas exchange, and it was agitated as above.

- Capmix agitation protocol:

The same procedure for vial preparation was followed as for tissue homogeniser agitation. Following gas exchange, the vial was placed in a Capmix agitator (Capmix Amalgam Capsule Mixing Machine, 3 M ESPE, Bayern, Germany) and agitated at the fixed setting of 4300 S/minute for 39 s. Prepared MBs were immediately stored on ice until further use.

- Sonication protocol:

The same phospholipid concentration as used in the agitation protocols was used for the sonication method in order to isolate the effect of the bubble formation step. 5 mL of the phospholipid suspension was transferred to a 7 mL glass vial (internal diameter × height: 9.5 × 60 mm). The tip of a sonicator probe (Q125, probe diameter 3 mm, 125 W, 20 kHz, QSonica, USA) was placed deep into the suspension and sonicated on 35% amplitude for 2.5 min (30 s on, 1 s off). Then, the vial headspace was flushed with PFB and the probe tip repositioned at the gas–liquid interface and further sonicated on 85% amplitude for 30 s, under constant gas flow. The vial was immediately capped and placed on ice.

### 2.3 Microbubble characterization and stability studies

For characterisation, MBs were transferred to a clean glass vial that was kept on ice, using an 18G needle and a 5 mL syringe while venting through another 18G needle. MB concentration and size distribution were measured using a Multisizer 4e (Beckman Coulter, Indianapolis, USA) with a 20 µm aperture. Three batches were prepared separately with each method.

### 2.4 Measurements of lipid order in the microbubble shell

C-laurdan is a photostable dye that shows spectral sensitivity to the environment polarity, characterized by a red shift in its emission spectrum in polar environments. It can be used to assess lateral organization of lipids in membranes[13]. The emission intensities at 440 and 490 nm can be used to calculate a generalized polarization (GP) value[14] which increases with increasing lipid order, reflecting the decrease in membrane water content.

In order to assess the lipid order in the MB shell, a previously established method was used with modifications [15], [16]. Briefly, C-laurdan was dissolved in DMSO at a final concentration of 1 mM. MBs were prepared fresh and washed once to remove residual lipids. MBs were loaded in a 5 mL syringe and spun gently on 300 g relative centrifugal force at 4 ⁰C for 5 min. The subnatant was removed by slowly depressing the plunger. The MB cake was redispersed back to 1 mL with PBS. For staining, MBs were diluted to a final concentration of 10^8^ particles/mL in PBS supplemented with 5 µL of the C-laurdan dye stock in a total volume of 500 µL (final C-laurdan concentration was 10 μM). MBs were incubated on ice for 30 min after adding the dye.

Imaging was performed on an inverted confocal microscope (LSM 780, Carl Zeiss Microscopy GmbH, Jena, Germany). 10 µL of stained MBs were deposited on a glass slide, covered with a cover-slip (0.09–0.13 mm thickness), and imaged with oil immersion 63x objective at the MB midplane. C-laurdan was excited with a 405 nm laser and emission was recorded between 433.8 and 442.7 nm (I_443_) and between 478.3 and 487.2 nm (I_487_) on a gallium arsenide phosphide spectral detector. All imaging was performed at room temperature.

Image processing was performed using a custom MATLAB script (R2022b, The MathWorks, Natick, Massachusetts, USA). Total fluorescence images for bubble segmentation were obtained as the sum of the two channels and pre-processed by linear intensity adjustment and self-guided filtering (neighbourhood size 11 × 11 pixels). An initial mask was obtained by binarization with Otsu's method and morphologically opened using a disk-shaped structuring element (3 pixels radius). The mask thus obtained was intersected with the initial mask to remove thin necks between adjacent bubbles, and hole-filled. A distance image was computed by applying a Euclidean distance transform to the inverted mask, followed by an H-minima transform of its negative (suppression depth 5) to prevent over-segmentation. All pixels of the distance image laying outside the mask were set to infinity and a watershed operation (8-connectivity) was then performed and all pixels outside the mask were set to zero to obtain regions corresponding to candidate bubbles. Candidates with a circularity below 0.5 or a convex area smaller than the area of a circle with a diameter of 1 μm were discarded. Each bubble was then analysed separately by cropping the image to the bounding box of the corresponding candidate region. Centre and radius were refined by applying a circular Hough transform, and a radial intensity profile within the corresponding circle was computed by averaging the intensities of all pixels at each distance from the center and smoothed by moving average (3 pixels window). Inner and outer shell radius were then computed as the first and last distance greater than 50% of the distance of maximum radial intensity. All further processing was performed on the raw images recorded by the microscope, without any pre-processing. Inner and outer shell radiuses were used to define a shell mask, which was further refined by excluding low-intensity pixels using the 75th percentile of pixel intensities outside the bubble as a threshold. Left and right truncation scores were calculated for each channel as the proportion of pixels intensities falling within a left or right boundary region with a boundary width proportional to the width of the intensity distribution (5% of the 10th-90th percentile range for n < 200 or 5% of the 5th-95th percentile range for n ≥ 200; widths were clipped to a minimum value of 0.005). Truncation of the intensity distribution in any channel would bias the GP distribution. Therefore, bubbles with truncation scores larger than 1.5% in any channel were discarded. Bubbles with shell thickness greater than 80% of the radius were also excluded from the analysis, as excessive shell thickness indicates that the focus is far from the equatorial plane and therefore the radius of the circle does not correspond to the radius of the bubble. For each remaining bubble, GP values were calculated for each pixel in the shell according to the equation

$$\mathrm{GP}=\frac{I_{443}-I_{487}}{I_{443}+I_{487}}$$

and used to compute the mean GP.

Three separate MB batches were prepared for each production method. The number of bubbles meeting the criteria for GP quantification varied between 88 and 1767 bubbles per batch.

### 2.5 Measurements of MB acoustic response

A High Intensity Focused Ultrasound (HIFU) transducer (H107 Sonic Concepts Inc, Bothell, Washington, USA) with a centre frequency of 0.5 MHz was used to stimulate MBs contained in a 2 mL Eppendorf tube, immersed in a degassed water bath at room temperature (∼21 °C). The transducer was driven at 0.5 MHz, 1% duty cycle and 5 Hz pulse repetition frequency (PRF) using a function generator (Agilent 33250A, Keysight Technologies Inc., California, USA) whose signal was amplified using a power amplifier (ENI 1040 L RF Amplifier, ENI, New York, USA). The peak negative pressure of the acoustic signal was varied from 0.2 to 2 MPa based on a calibration performed with a needle hydrophone (hydrophone was placed at the ultrasound focus and 24 voltages applied to generate transducer peak negative voltages that were recorded and converted to peak negative pressures). MBs were diluted to a final concentration of 10^8^ particles/mL in 2 mL total volume, transferred to a 2 mL Eppendorf tube, and exposed to ultrasound for 5 min. For each measurement a separate aliquot of MBs was used. The acoustic emissions from the MBs were recorded with an unfocused transducer with 5 MHz centre frequency (Olympus V309-SU, Evident Europe GmbH, Stansted, UK). The unfocused transducer was integrated coaxially into a cut-out in the HIFU transducer. The signal from this transducer was filtered through a 1.8 MHz high-pass filter used to remove the drive frequency, amplified (SR445A, SRS, Sunnyvale, CA) and then digitised with a digital oscilloscope (Handyscope HS3, TiePie Engineering, Sneek, Netherlands) before further analysis.

A second custom MATLAB script was used to analyse the acoustic emissions. First, a Hamming window was applied to the time domain signal and power spectral density was estimated with Welch’s-method [17]. The power of the acoustic emissions was calculated for each pulse and the total energy was calculated over the entire exposure time. For each pulse, cavitation was deemed to occur if the total power was >30-fold (14.8 dB) higher than the noise floor recorded before the function generator was switched on. Cavitation time was calculated as a sum of the lengths of pulses satisfying this condition. Corresponding mean power was also calculated as mean power over the period where cavitation was detected.

### 2.6 Statistical analysis

All experiments were repeated with three separate MB batches for each manufacturing method compared. Descriptive statistics were calculated in R (version 4.0.0) and used to summarise data; results are reported as mean values and standard deviation.

## 3 Results and discussion

### 3.1 Microbubble size and concentration

**Fig. 1:**
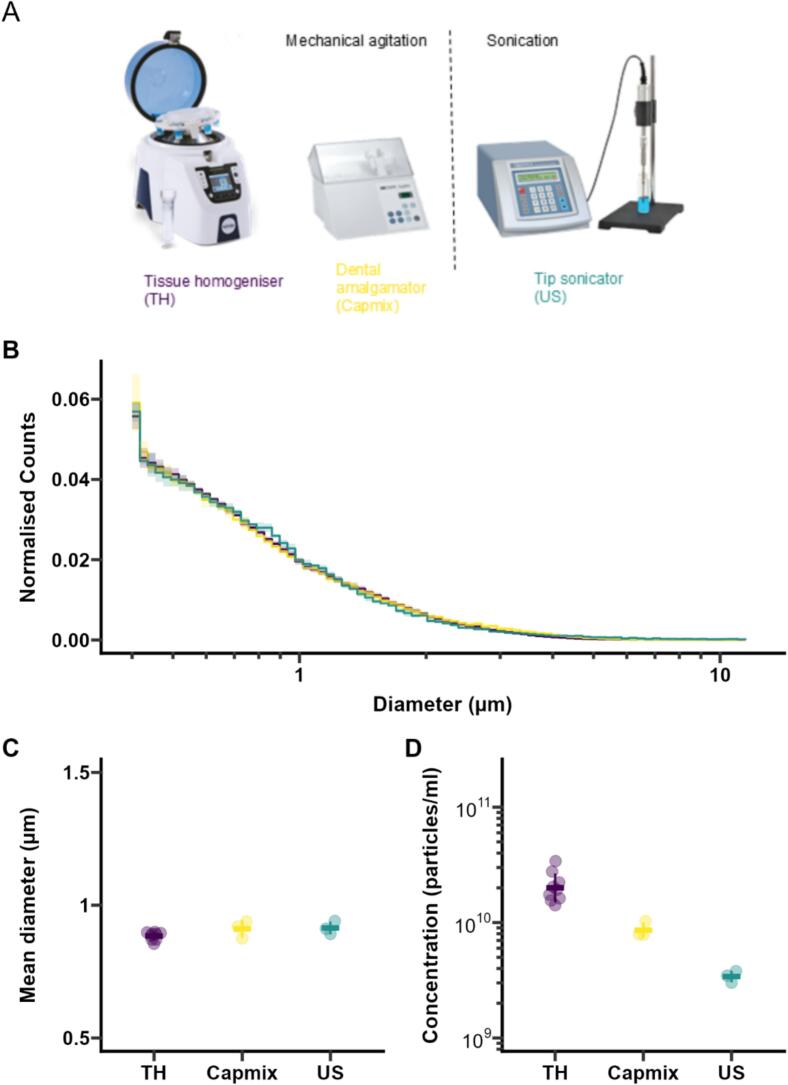
Size characterisation of MBs prepared with three different methods. (A) Methods for microbubble production. (B) Size distribution (solid line represents mean of 3 batches, shaded area represents ± SD). (C) Concentration (error bars represent standard deviation). (D) Mean diameter (error bars represent standard deviation). (B-D) are obtained from Multisizer measurements.

The size distributions of the MBs prepared by the three different methods (Fig. 1B) were almost identical, with a mean diameter^1^ of 0.91 ± 0.02 µm (n = 3 batches), 0.91 ± 0.03 µm (n = 3 batches) and 0.88 ± 0.02 µm (n = 9 batches) for MBs prepared by sonication, Capmix and tissue homogeniser, respectively (Table 1, Fig. 1C). MBs prepared by sonication had a slightly broader size distribution with an average 87% coefficient of variation, compared to MBs prepared by Capmix and tissue homogeniser that had coefficients of variation of 76% and 72%, respectively. The fraction of particles in the clinically relevant range was also very similar, with 25%, 27% and 26% particles larger than 1 µm for MBs prepared by sonication, Capmix and tissue homogeniser, respectively, and <1% particles larger than 5 µm for all three methods (Supp. Fig. S1C). In order to provide a fair comparison to commercially available contrast agents, the microbubbles were not washed before characterisation, as the conditions required to wash microbubbles in this size range (centrifugation at 300 g) may affect their size distribution [18]. Residual lipid structures, which are present in a concentration that is approximately independent of MB concentration, are therefore also counted when measuring MB size and concentration, which may lead to underestimating the mean diameter at low MB concentrations. The 90th percentile of the distribution (D_90_), a statistic less affected by the presence of residual lipids, was also comparable, with values of 1.55 ± 0.06 µm, 1.64 ± 0.11 µm and 1.57 ± 0.05 µm for MBs prepared by sonication, Capmix and tissue homogeniser, respectively (Supp. Fig. S1B). A large difference was however observed in the concentration measurements: the concentration of MBs was ∼ 10^10^ particles/mL for both agitation methods (Fig. 1D), while sonication produced a lower concentration by an order of magnitude (∼10^9^ particles/mL). This may have been due to the phospholipid concentration (0.9 mg/mL) being lower than that typically used for sonication (>4 mg/mL). While it is possible to obtain higher microbubble concentrations by increasing the phospholipid concentration, the trade-off is a correspondingly higher cost per batch and a larger excess of unembedded phospholipids, which can be problematic for further functionalisation. The batch-to-batch variation was similar for all methods, despite the sensitivity of the sonication protocol to process parameters that are difficult to standardise accurately with standard laboratory equipment, including gas flow rate and positioning of the ultrasound probe and gas outlet.

**Table 1:** Particle size measurements of MBs produced by different methods, obtained with a Multisizer 4e. ^a^ Coefficient of variation of the size distribution; ^b^ 90th percentile diameter; ^c^ Fraction of bubbles larger than 1 µm; ^d^ Fraction of bubbles larger than 5 µm.

Method	Batch	D[1] (µm)	CV (%)^a^	D_90_ (µm)^b^	P(D > 1 µm) (%)^c^	P(D > 5 µm) (%)^d^	Concentration (particles/ml)	Gas volume (µl/ml)
Capmix	1	0.92	75	1.68	26.8	0.2	7.9 × 10^9^	12.7
	2	0.94	75	1.72	28.9	0.2	1.0 × 10^10^	18.6
	3	0.87	77	1.52	24.4	0.3	7.9 × 10^9^	13.9
	*Mean (SD)*	*0.91 (0.03)*	*76 (1)*	*1.64 (0.11)*	*26.7 (2.2)*	*0.2 (0.0)*	*8.7 × 10^9^ (1.4 × 10^9^)*	*15.0 (3.1)*

TH	1	0.88	73	1.61	25.0	0.2	1.4 × 10^10^	20.7
	2	0.90	69	1.64	26.8	0.1	1.6 × 10^10^	20.4
	3	0.90	65	1.60	27.5	0.1	2.0 × 10^10^	23.3
	4	0.87	73	1.53	25.2	0.2	2.2 × 10^10^	35.4
	5	0.85	78	1.48	23.9	0.3	3.4 × 10^10^	62.9
	6	0.89	76	1.57	26.6	0.3	2.8 × 10^10^	51.1
	7	0.90	76	1.60	26.3	0.3	1.7 × 10^10^	31.3
	8	0.89	69	1.57	26.7	0.2	1.9 × 10^10^	25.7
	9	0.87	72	1.53	24.9	0.2	1.6 × 10^10^	23.2
	*Mean (SD)*	*0.88 (0.02)*	*72 (4)*	*1.57 (0.05)*	*25.9 (1.2)*	*0.2 (0.1)*	*2.1 × 10^10^ (6.4 × 10^9^)*	*32.7 (14.9)*
US	1	0.94	89	1.61	26.9	0.8	3.5 × 10^9^	10.6
	2	0.91	90	1.55	24.6	0.8	3.0 × 10^9^	8.8
	3	0.89	82	1.49	24.8	0.5	3.8 × 10^9^	8.5
	*Mean (SD)*	*0.91 (0.02)*	*87 (4)*	*1.55 (0.06)*	*25.4 (1.3)*	*0.7 (0.2)*	*3.4 × 10^9^ (3.9 × 10^8^)*	*9.3 (1.1)*

### 3.2 Shell lipid order

**Fig. 2:**
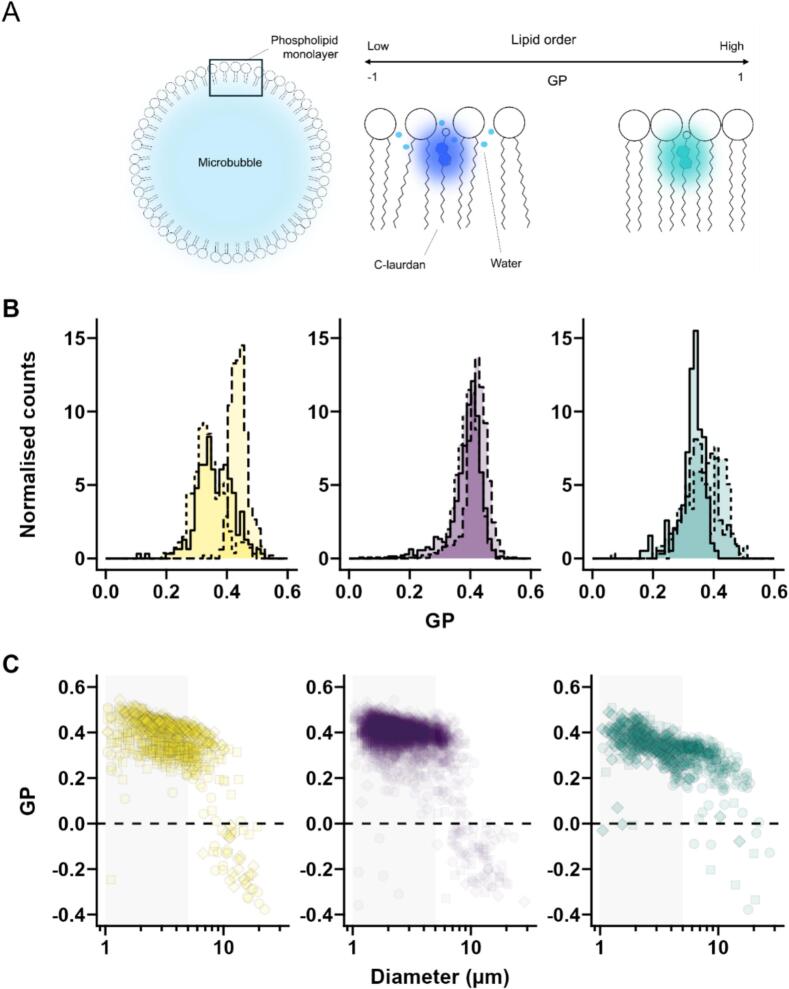
Schematic representation of the relationship between C-laurdan emission and monolayer fluidity (A); Plot of GP distributions (B) and GP values versus mean diameter (C) in MBs prepared by tissue homogeniser Capmix and sonication. GP distributions refer to MBs in the clinical range (1–5 μm), shown in grey. Different point shapes (circle, square and diamond) and line styles (solid, dashed, dotted) represent different batches.

Shell properties, including surface tension, are important parameters in determining the response of microbubbles to ultrasound fields. MB production methods were therefore compared using generalised polarisation as a qualitative measure of surface property differences [19]. The lipid order of the MB shell and its size dependence were investigated with C-laurdan staining (Fig. 2). MBs prepared by all three methods showed a similar trend – the smaller, more clinically relevant MBs (1–5 µm) had a higher lipid order than the larger MBs. This phenomenon was observed particularly in the MBs produced with the tissue homogeniser. MBs larger than ∼7 µm in diameter showed a clear decrease in GP with increasing size. Above ∼10 µm, all measured MBs had a negative GP value, characteristic of a liquid-disordered (Lα) phase (Fig. 2A). Capmix-produced MBs exhibited the same GP inversion trend (Fig. 2C), but large MBs produced by sonication did not. Instead, the majority of sonicated MBs larger than 10 µm in diameter had similar GP values to the smaller MBs. This suggests that the larger MBs produced by sonication might be more stable than those produced by agitation; and might partially explain why sonication produced higher concentrations of large MBs (0.7% >5 µm) when compared to the agitation methods (0.2% for both Capmix and tissue homogeniser, Supp. Fig. S1C).

In the clinically relevant MB size range, the mean of the GP distribution was similar for Capmix (0.38 ± 0.05, n = 3 batches) and tissue homogeniser (0.40 ± 0.02, n = 3 batches), and slightly lower for sonication (0.35 ± 0.02, n = 3 batches). (Fig. 2B, Supp. Table S1). The batch-to-batch variation was ∼5% for both tissue homogeniser and sonication, while the batches produced with the Capmix were less reproducible, with a batch-to-batch variation of 14%.

### 3.3 Effect of varying processing parameters

The formation of MBs in a bulk lipid suspension whether by sonication or agitation, is a complex process. Mechanistically, it has been proposed that gas pockets form on the gas/liquid interface and are pushed into the liquid where they gain the stabilizing lipid layer either from the lipids present on the interface or those transferred from precursor lipid vesicles. These lipids stabilize the gas cores via adsorption, forming coated MBs [20], [21]. Therefore, it was hypothesized that varying the time or frequency of agitation, and subsequently shear mixing, would have an impact on both MB concentration and size.

**Fig. 3:**
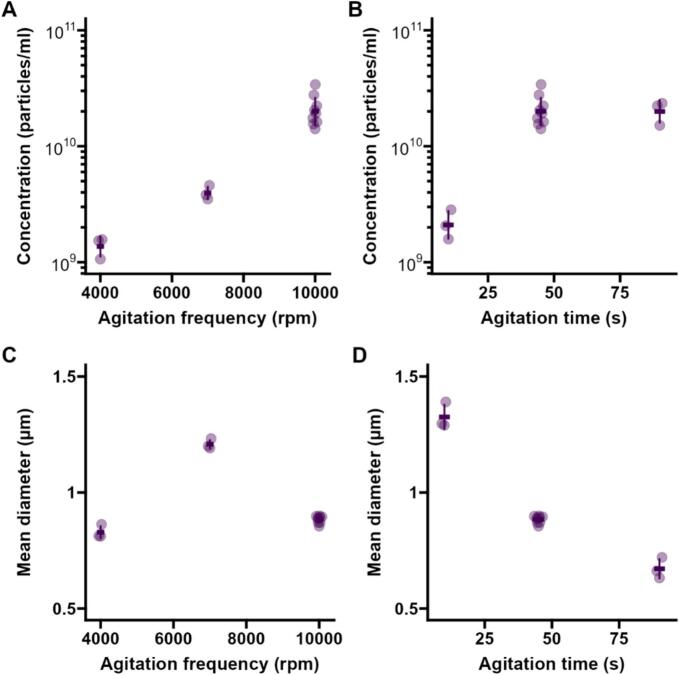
Effect of agitation frequency (A–C) and agitation time (B–D) on concentration (A–B) and mean size (C–D) of MBs produced with the tissue homogeniser.

Fig. 3B and D show the effect on MB size and concentration of varying the agitation time, while keeping the agitation frequency constant at 10000 cpm. Increasing the agitation time from 10 to 45 s led to a 10-fold increase in MB concentration and a decrease in mean size (Supp. Table S2). A further increase in agitation time to 90 s did not appreciably influence MB size or concentration. Next, the frequency of agitation was varied while keeping the time fixed at 45 s (Fig. 3C and D, Supp. Table S3). Increasing the frequency from 4500 cpm to 10000 cpm (the maximum frequency achievable with the tissue homogeniser used for this work) resulted in an exponential increase in MB concentration (Fig. 3A). The mean size increased with increasing frequency initially, and then decreased (Fig. 3C).

These results can be at least partially explained by considering the turbulent conditions during the agitation process. As the stroke length (3.4 cm) is comparable to the vial dimensions, the liquid impinges on the walls twice per cycle at high velocity (up to ∼18 m/s at 10000 cpm), generating splashing and re-entrant jets which entrain the gas phase. Chaotic advection in this fully turbulent flow (Reynolds number ∼10^5^) stretches and folds the entrained gas pockets into thread-like regions (ligaments) which are then pinched-off by capillary instabilities to form bubbles. It is reasonable to expect the number of sites available for bubble formation to scale with gas–liquid interfacial area, which in this regime grows exponentially with the shear rate $\dot{\gamma }$. Since $\dot{\gamma }$ scales as $\sqrt{∊}$, where ∊ is the energy dissipation rate, and ∊ is in turn proportional to the cube of velocity (and therefore of shaking frequency f), this would result in an exponential dependence of bubble concentration on ${\dot{\gamma }\propto f}^{1.5}$, which is consistent with our data [20]. In this simplified picture, bubble formation or fragmentation occur when a ligament or a larger bubble stretch to a diameter d that causes the capillary number (the ratio of viscous stress $\mu \dot{\gamma }$ and interfacial stress σ/d), to drop below a critical threshold.^2^ The inverse dependence on $\dot{\gamma }$ would then lead to a frequency-dependent maximum diameter ($d\propto f^{-1.5}$), which is in turn proportional to the mean diameter [16]. The measured mean bubble diameter does indeed decrease with frequency between 7000 cpm and 10000 cpm, but it increases between 4500 cpm and 7000 cpm. This anomalous trend may be partially explained by the higher lipid-to-microbubbles ratio at low microbubbles concentration, which may cause underestimation of the mean diameter due to residual lipid structures (see Microbubble Size and Concentration). Other frequency-dependent factors may also contribute: the simple *f*^−1.5^ scaling does not take into account thermal effects and the relative timescales of interfacial area growth and surfactant adsorption. Both effects would hinder bubble fragmentation as the frequency increases: the former by reducing viscosity, the latter by reducing surface coverage and therefore increasing the effective surface tension.

At a fixed frequency, MB concentration increased initially with prolonging the agitation time from 10 to 45 s, while the mean diameter decreased. The decrease in size is consistent with fragmentation of existing large MBs into smaller bubbles. However, further increase in agitation time to 90 s did not result in drastic changes of either concentration or size, indicating that the system may have reached a steady state. This suggests that the rates of bubble formation and disappearance may be affected by the MB concentration. The lack of further MB fragmentation after the 45 s timepoint is also consistent with a frequency-dependent limiting size dictated by the balance of viscous stress and interfacial stress, as discussed above. Thermal effects may also have played a role: while initial and final temperatures were strictly controlled by storing the lipid solution on ice immediately before and immediately after agitation, high-speed agitation of similar formulations at 4500 cpm for 45 s is known to cause substantial transient heating [22], which may increase further with agitation time.

### 3.4 Effect of batch volume

**Fig. 4:**
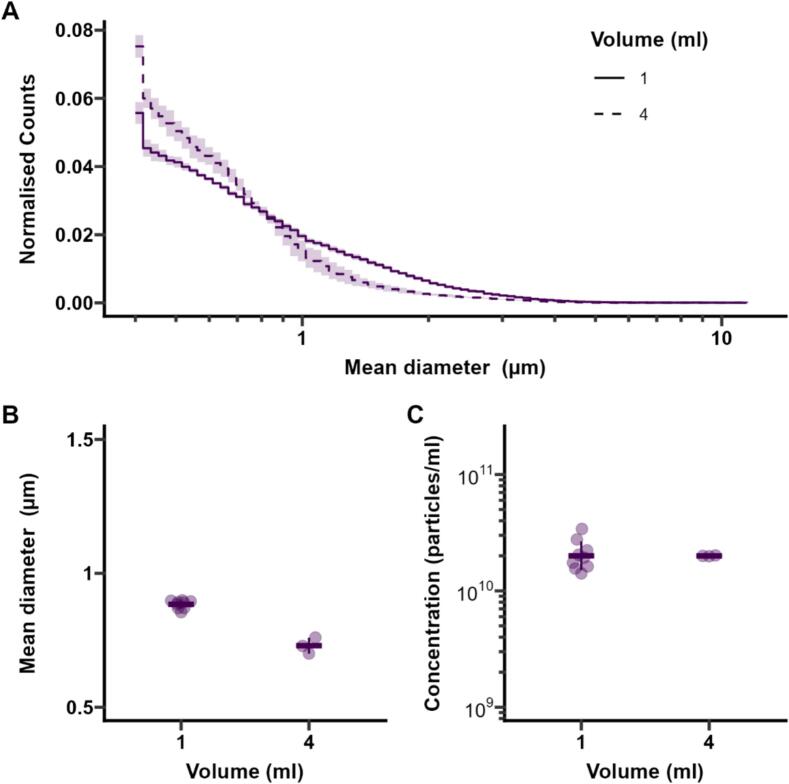
Normalised size distributions (A), concentration (B) and mean diameter (C) of MBs prepared in a large vial (4 mL of suspension) compared with MBs prepared in a standard small vial (1 mL of suspension).

One of the shortcomings of dental amalgamators is their inability to produce MB volumes larger than 1 mL due to the limit of the vial size that can fit into the agitator. To overcome this, a scale up to 4 mL was investigated using the tissue homogeniser with a larger vial. On-demand bubble manufacturing of larger batches may be beneficial in both clinical settings, where up to three vials of Definity have been administered to individual patients in clinical trials [23], [24], and research settings, where larger batches could help eliminate batch-to-batch variation in controlled experiments. In this study, scale up from 1 to 4 mL was investigated using the tissue homogeniser with a larger vial. MBs were produced by using the same protocol as for the standard small vial (1 mL of suspension) and were shown to have similar size distribution, with both mean diameter (fold change 0.82) and concentration (fold change 0.96) remaining within 20% of those produced in a small vial (Fig. 4A and B, Supp. Table S4). The differences observed in the size distribution are likely due to different geometry of the vial and the liquid column, which is expected to affect the fluid motion during shaking and therefore the MB parameters.

### 3.5 Ultrasound response

**Fig. 5:**
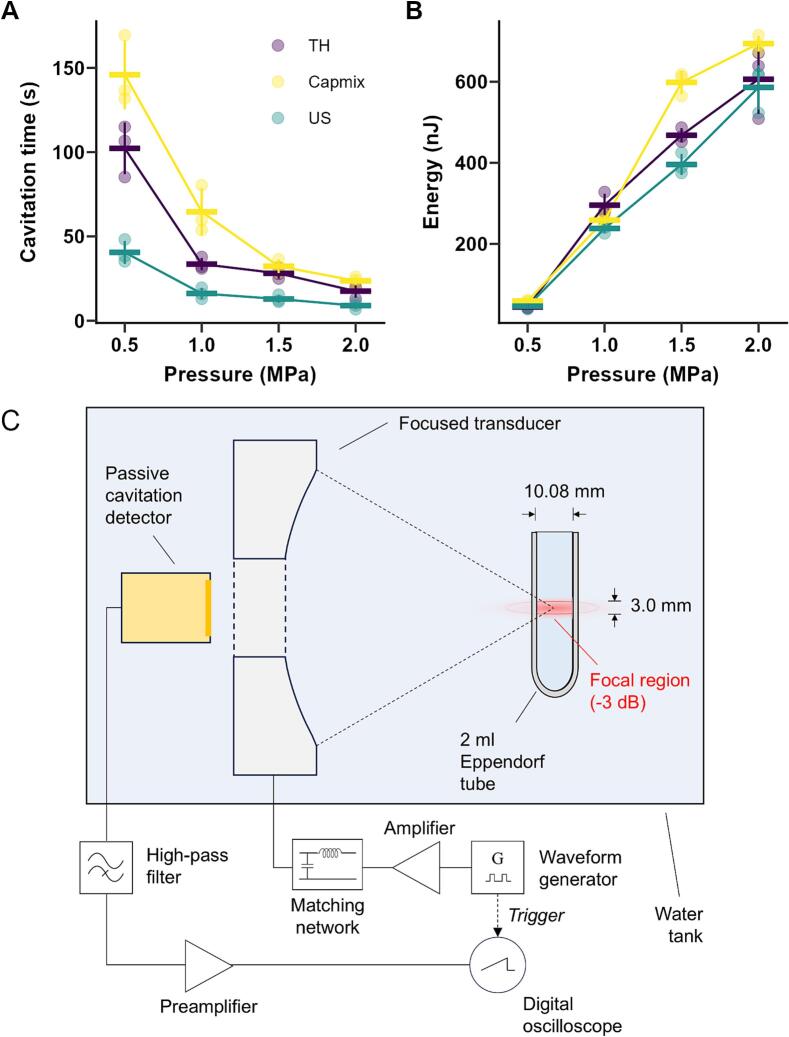
Response of MB produced by different methods to 0.5 MHz ultrasound exposure (1% DC, 5 Hz PRF) at different peak negative pressures in terms of (A) cavitation time and (B) total energy of acoustic emissions. A concentration of 10^8^ MBs/mL was used in all experiments. (C) A schematic of the experimental setup used for the ultrasound exposure of the MBs.

As MB size and structure will affect bubble dynamics, the effect of manufacturing method on acoustic response was investigated. The acoustic response of the MBs prepared by the three different methods was measured by exposing them to pulses of ultrasound (0.5 MHz centre frequency, 1% duty cycle, 5 Hz PRF) with different peak negative pressures, over 2 min, at a matched concentration of 10^8^ particles/mL. The corresponding acoustic emissions were recorded and used to estimate broadband power for each pulse and cavitation time, defined as the period of time over which power was more than 30-fold higher (14.8 dB) than the noise floor. An increase in the total energy of acoustic emissions was recorded for all samples with increasing peak negative pressure, as expected, and the cavitation time decreased. No substantial difference was observed between MBs prepared by agitation methods in either cavitation time (Fig. 5A) or energy of acoustic emissions (Fig. 5B). Both methods outperformed MBs produced by sonication in cavitation time, by showing a more persistent response and cavitating longer, with a fold increase over sonication of 2.0 to 2.5 for tissue homogeniser and 2.7 to 4 for Capmix. The suspension was optically clear at the end of every treatment, indicating that the bubbles were well-mixed and confirming that signal disappearance reflects complete dissolution rather than creaming. The total energy of acoustic emissions was similar for all methods at all driving pressures, with absolute fold changes <1.5 with respect to sonication.

## 4 Conclusions

Here, we compared different methods for the production of phospholipid-coated MBs. Two advantages of using agitation over sonication for MB production are that it requires a lower lipid concentration, and it does not require a probe to be placed in the lipid suspension, which could contaminate the sample. Moreover, it is much easier to implement in both laboratory and clinical environments, requiring minimal training of staff and easy to use equipment. Currently, dental amalgamators have limited utility as they can produce only one sample at a time, are not suitable for producing volumes larger than 1 mL and are prone to mechanical failure. Our aim was to overcome these limitations by producing MBs of similar characteristics by employing a bead mill homogeniser, a type of instrument commonly used in both research and clinical laboratories. We have shown that MBs produced using this method have a similar size distribution and concentration as those produced with a dental amalgamator and by sonication. MB shell lipid order was also investigated and compared, and the new method was found to produce MBs with slightly higher lipid order than those produced using sonication and with lower batch-to-batch variation than those produced with a dental amalgamator. We also optimised examined the effect of agitation frequency and time on MB formation, and found a set of parameters that generate high MB concentrations (2 × 10^10^ particles/mL) whilst retaining a clinically relevant size distribution (∼25% of bubbles in the 1–5 μm range). The tissue homogeniser method was successfully scaled up by four times (from 1 to 4 mL) with minimal changes in either mean diameter or concentration. We tested MB ultrasound response and found no substantial difference in cavitation time or acoustic emissions from MBs prepared by either agitation method, both of which outperformed MBs prepared by sonication by cavitating for a longer time, especially at low driving pressures.

In summary, our results indicate that MBs produced with a tissue homogeniser have comparable properties to those produced with agitators used in the clinic, while offering significant advantages in terms of scalability.

## CRediT authorship contribution statement

**Jovana Katrinka-Mavrak:** Writing – original draft, Visualization, Investigation. **Luca Bau:** Writing – review & editing, Supervision, Methodology, Investigation. **Jocelyne Rivera:** Writing – review & editing, Methodology. **Adam C. Sedgwick:** Writing – review & editing, Supervision. **Eleanor Stride:** Writing – review & editing, Supervision, Project administration, Funding acquisition, Conceptualization.

## Declaration of competing interest

The authors declare the following financial interests/personal relationships which may be considered as potential competing interests: Eleanor Stride reports a relationship with SonoTarg Ltd that includes: equity or stocks. If there are other authors, they declare that they have no known competing financial interests or personal relationships that could have appeared to influence the work reported in this paper.

## Appendix A Supplementary data

Supplementary Data 1 • Table S1. Summary statistics of GP distributions for MBs produced with the three methods. • Table S2. Size distribution statistics of MBs produced with a tissue homogeniser at different agitation times. • Table S3. Size distribution statistics of MBs produced with a tissue homogeniser at different agitation frequencies. • Table S4. Size distribution statistics of MBs produced with a tissue homogeniser at different scale. • Figure S1. Log-transformed normalised size distributions (A), 90th percentile diameter (B) and fraction of bubbles larger than 5 μm (C) for MBs produced with the three methods. • Figure S2. Normalised size distributions of MBs produced with a tissue homogeniser at (A) different agitation frequencies and (B) different agitation times • Figure S3. 3D model of the adaptor used for inserting small vials into the Precellys Evolution tissue homogeniser.

## Footnotes

1 Particles smaller than 400 nm in diameter were not measured; the term particle is used here as the instrument used for the size distribution measurements cannot distinguish between MBs and other particles.

2 We assume that bubble fragmentation occurs because of viscous, rather than inertial, stress, as virtually all bubbles are smaller than the Kolmogorov scale (∼20 μm in our conditions). Nevertheless, similar arguments apply to the inertial case, where the maximum stable diameter is determined by the balance between inertial and interfacial stress, leading to a similar frequency dependence ($f^{-1.2}$).
