# Supplementary material for: Comparison of ultrasound contrast agent production by sonication and mechanical agitation
Source: Ultrason Sonochem. 2026 May 6;129:107875. doi: 10.1016/j.ultsonch.2026.107875 (PMC13191645; doi:10.1016/j.ultsonch.2026.107875)

| Method | Batch | n^†^ | GP^††^ | CV^†††^ (%) |
| --- | --- | --- | --- | --- |
| TH | 1 | 1056 | 0.38 | 19 |
|  | 2 | 1329 | 0.39 | 10 |
|  | 3 | 1767 | 0.42 | 9 |
|  | Mean ± SD |  | 0.40 ± 0.02 | 13 ± 5 |
| Capmix | 1 | 230 | 0.36 | 17 |
|  | 2 | 200 | 0.34 | 20 |
|  | 3 | 359 | 0.44 | 6 |
|  | Mean ± SD |  | 0.38 ± 0.05 | 14 ± 7 |
| Sonication | 1 | 142 | 0.33 | 14 |
|  | 2 | 88 | 0.37 | 20 |
|  | 3 | 456 | 0.36 | 19 |
|  | Mean ± SD |  | 0.35 ± 0.02 | 17 ± 3 |

Supplementary Table S1: Summary statistics of GP distributions for MBs produced with the three methods. ^†^ Number of bubbles analysed. ^††^Mean of the GP distribution. ^†††^Coefficient of variation of the GP distribution. All distributions are for the population of MBs in the clinical range (1-5 μm).

| Time (s) | n | D[1,0] (µm)^a^ | CV (%)^a^ | D_90_ (µm)^b^ | P(D > 1 µm) (%)^c^ | P(D > 5 µm) (%)^d^ | Concentration (particles/ml) | Gas volume (µl/ml) |
| --- | --- | --- | --- | --- | --- | --- | --- | --- |
| 10 | 3 | 1.33 (0.06) | 105 (6) | 2.83 (0.15) | 40.3 (2.0) | 2.6 (0.6) | 2.2 × 10^9^ (6.3 × 10^8^) | 21.4 (5.3) |
| 45 | 9 | 0.88 (0.02) | 72 (4) | 1.57 (0.05) | 25.9 (1.2) | 0.2 (0.1) | 2.1 × 10^10^ (6.4 × 10^9^) | 32.7 (14.9) |
| 90 | 3 | 0.67 (0.05) | 62 (7) | 0.90 (0.07) | 7.0 (2.1) | 0.1 (0.1) | 2.0 × 10^10^ (4.5 × 10^9^) | 14.8 (9.4) |

Supplementary Table S2: Particle size measurements of MBs produced with a tissue homogeniser at different agitation times, obtained with a Multisizer 4e. ^a^ Coefficient of variation of the size distribution; ^b^ 90th percentile diameter; ^c^ Fraction of bubbles larger than 1 µm; ^d^ Fraction of bubbles larger than 5 µm.

| Frequency (cpm) | n | D[1,0] (µm) | CV (%)^a^ | D_90_ (µm)^b^ | P(D > 1 µm) (%)^c^ | P(D > 5 µm) (%)^d^ | Concentration (particles/ml) | Gas volume (µl/ml) |
| --- | --- | --- | --- | --- | --- | --- | --- | --- |
| 10000 | 9 | 0.88 (0.02) | 72 (4) | 1.57 (0.05) | 25.9 (1.2) | 0.2 (0.1) | 2.1 × 10^10^ (6.4 × 10^9^) | 32.7 (14.9) |
| 7000 | 3 | 1.21 (0.02) | 95 (4) | 2.39 (0.05) | 39.5 (0.7) | 1.6 (0.2) | 4.0 × 10^9^ (5.6 × 10^8^) | 25.6 (7.9) |
| 4500 | 3 | 0.83 (0.03) | 106 (4) | 1.48 (0.10) | 17.8 (1.2) | 0.8 (0.1) | 1.4 × 109 (2.8 × 10^8^) | 4.4 (0.5) |

Supplementary Table S3: Particle size measurements of MBs produced with a tissue homogeniser at different agitation frequencies, obtained with a Multisizer 4e. ^a^ Coefficient of variation of the size distribution; ^b^ 90th percentile diameter; ^c^ Fraction of bubbles larger than 1 µm; ^d^ Fraction of bubbles larger than 5 µm.

| Volume (ml) | n^a^ | D[1,0] (µm) | CV (%)^a^ | D_90_ (µm)^b^ | P(D > 1 µm) (%)^c^ | P(D > 5 µm) (%)^d^ | Concentration (particles/ml) | Gas volume (µl/ml) |
| --- | --- | --- | --- | --- | --- | --- | --- | --- |
| 1 | 9 | 0.88 (0.02) | 72 (4) | 1.57 (0.05) | 25.9 (1.2) | 0.2 (0.1) | 2.1 × 10^10^ (6.4 × 10^9^) | 32.7 (14.9) |
| 4 | 3 | 0.73 (0.03) | 66 (5) | 1.12 (0.10) | 13.8 (3.4) | 0.1 (0.1) | 2.0 × 10^10^ (1.7 × 10^8^) | 16.6 (2.9) |

Supplementary Table S4: Particle size measurements of MBs produced with a tissue homogeniser at different scale, obtained with a Multisizer 4e. ^a^ Coefficient of variation of the size distribution; ^b^ 90th percentile diameter; ^c^ Fraction of bubbles larger than 1 µm; ^d^ Fraction of bubbles larger than 5 µm.


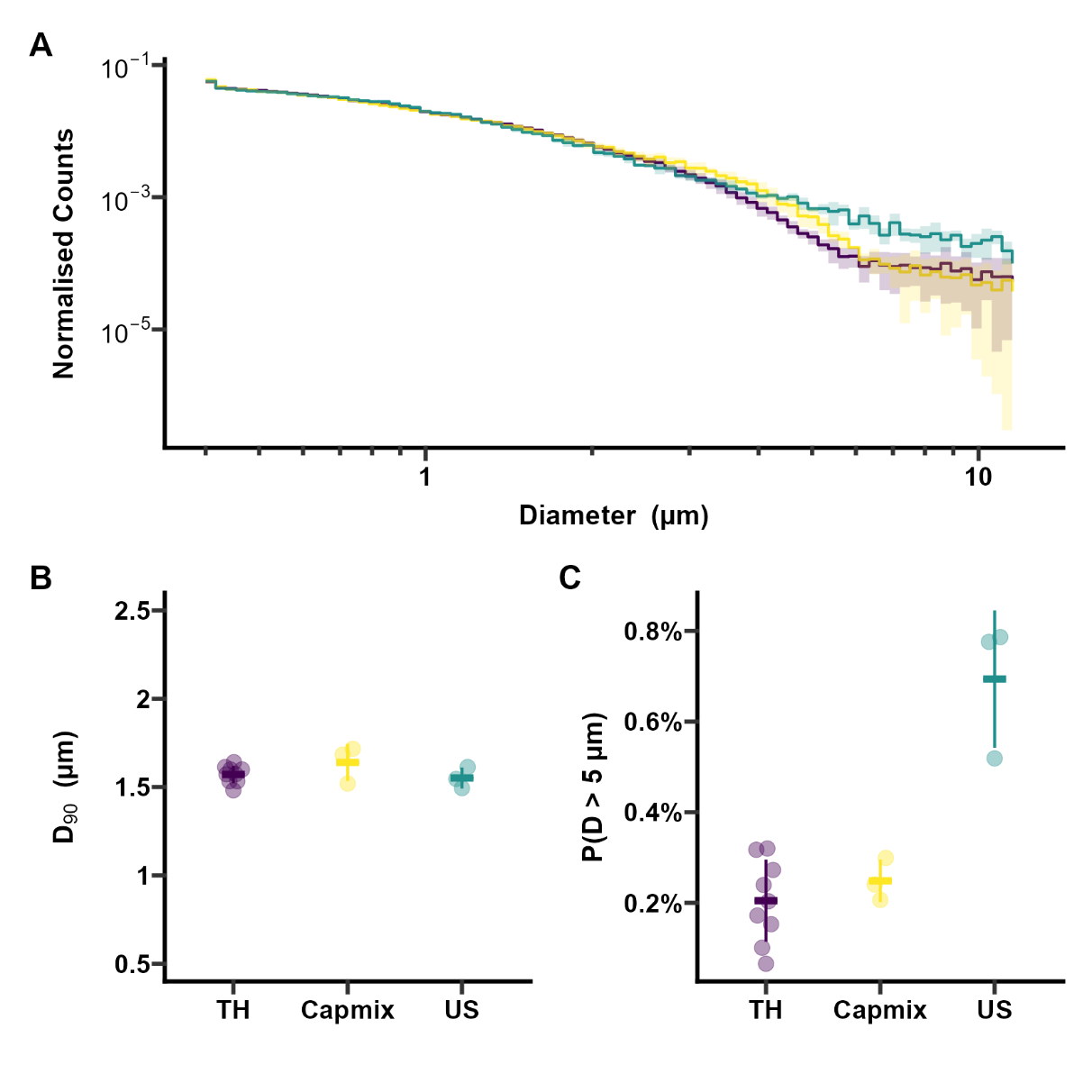


Supplementary Figure S1: (A) Log-transformed normalised size distributions, (B) 90^th^ percentile diameter of the bubble size distribution and (C) fraction of bubbles larger than 5 μm for MBs produced with different methods. All size distributions were obtained from Multisizer measurements.


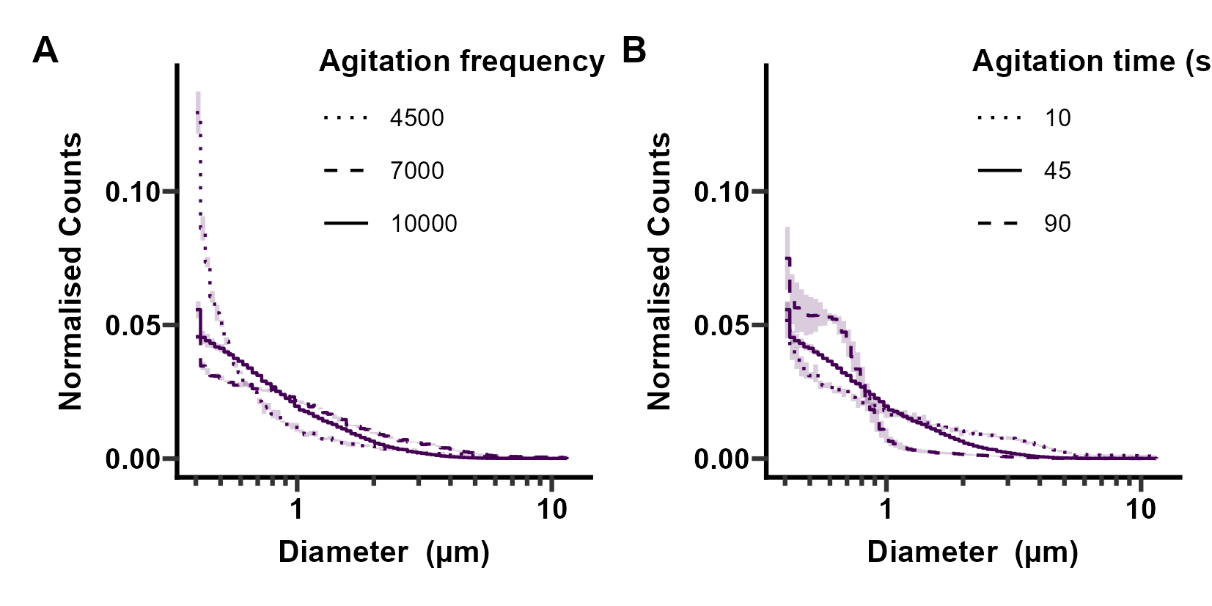


Supplementary Figure S2: Normalised size distributions of MBs produced with a tissue homogeniser at (A) different agitation frequencies and (B) different agitation times. All size distributions were obtained from Multisizer measurements.

Supplementaty Figure S3: 3D model of the adaptor used for inserting small vials into the Precellys Evolution tissue homogeniser.


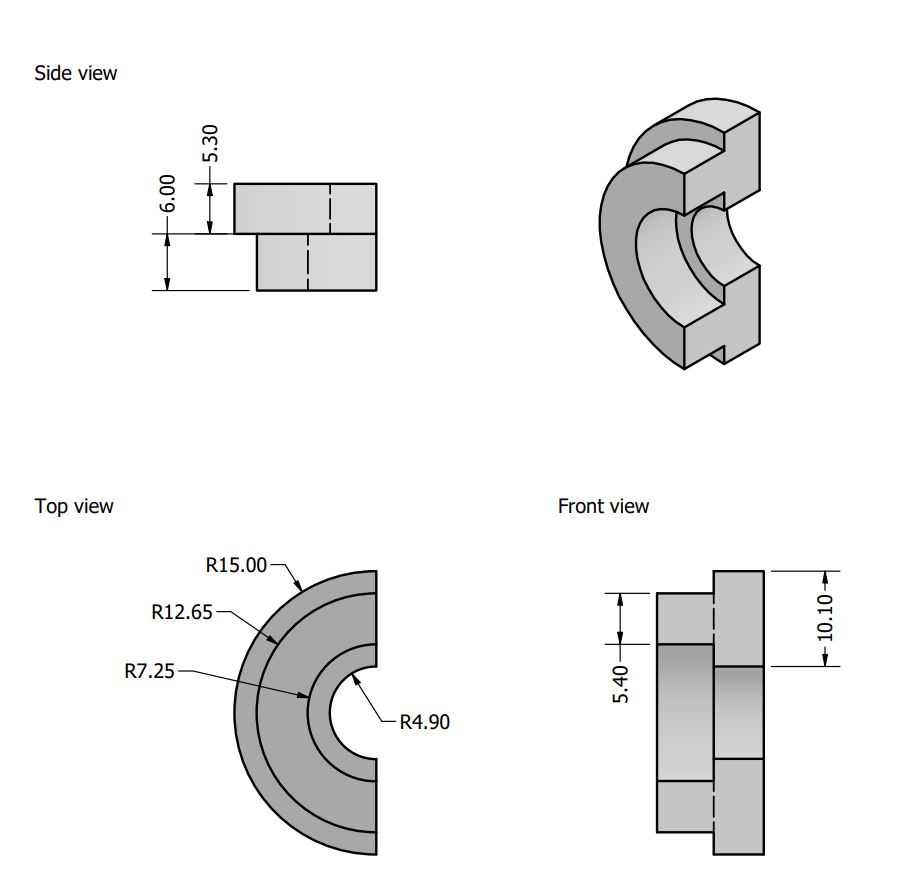

Supplement: Supplementary Data 1 — • Table S1. Summary statistics of GP distributions for MBs produced with the three methods. • Table S2. Size distribution statistics of MBs produced with a tissue homogeniser at different agitation times. • Table S3. Size distribution statistics of MBs produced with a tissue homogeniser at different agitation frequencies. • Table S4. Size distribution statistics of MBs produced with a tissue homogeniser at different scale. • Figure S1. Log-transformed normalised size distributions (A), 90th percentile diameter (B) and fraction of bubbles larger than 5 μm (C) for MBs produced with the three methods. • Figure S2. Normalised size distributions of MBs produced with a tissue homogeniser at (A) different agitation frequencies and (B) different agitation times • Figure S3. 3D model of the adaptor used for inserting small vials into the Precellys Evolution tissue homogeniser. [file mmc1.docx]
